# Extracellular synthesis of zinc oxide nanoparticle using seaweeds o*f* gulf of Mannar*, India*

**DOI:** 10.1186/1477-3155-11-39

**Published:** 2013-12-03

**Authors:** Sangeetha Nagarajan, Kumaraguru Arumugam Kuppusamy

**Affiliations:** 1 Department of Marine and Coastal Studies, Madurai Kamaraj University, Madurai, Tamil Nadu 21, India

**Keywords:** Biosynthesis, Gulf of Mannar, Characterization, Seaweeds, Zinc oxide

## Abstract

**Background:**

The biosynthesis of metal nanoparticles by marine resources is thought to be clean, nontoxic, and environmentally acceptable “green procedures”. Marine ecosystems are very important for the overall health of both marine and terrestrial environments. The use of natural sources like Marine biological resources essential for nanotechnology. Seaweeds constitute one of the commercially important marine living renewable resources. Seaweeds such as green *Caulerpa peltata*, red *Hypnea Valencia* and brown *Sargassum myriocystum* were used for synthesis of Zinc oxide nanoparticles.

**Result:**

The preliminary screening of physico-chemical parameters such as concentration of metals, concentration of seaweed extract, temperature, pH and reaction time revealed that one seaweed *S. myriocystum* were able to synthesize zinc oxide nanoparticles. It was confirmed through the, initial colour change of the reaction mixture and UV visible spectrophotometer. The extracellular biosynthesized clear zinc oxide nanoparticles size 36 nm through characterization technique such as DLS, AFM, SEM –EDX, TEM, XRD and FTIR. The biosynthesized ZnO nanoparticles are effective antibacterial agents against Gram-positive than the Gram-negative bacteria.

**Conclusion:**

Based on the FTIR results, fucoidan water soluble pigments present in *S. myriocystum* leaf extract is responsible for reduction and stabilization of zinc oxide nanoparticles. by this approach are quite stable and no visible changes were observed even after 6 months. These soluble elements could have acted as both reduction and stabilizing agents preventing the aggregation of nanoparticles in solution, extracellular biological synthesis of zinc oxide nanoparticles of size 36 nm.

## Background

Nanotechnology is emerging as a rapidly growing field with its application in Science and Technology for the purpose of manufacturing new materials at the nanoscale level [1]. The biologically diverse marine environment has a great promise for nanoscience and nanotechnology. Biosynthetic and environment friendly technology for the synthesis of zinc oxide (ZnO) NPs are believed to be nontoxic, biosafe, and biocompatible and have been used as drug carriers, cosmetics, and fillings in medical materials [2]. However most ZnO nano-particles used commercially produced synthetically which have some advantages, compared to silver nano-particle, such as lower cost, white appearance [3].

Chemical and physical methods of synthesis are costly and require extensive labour and time. Furthermore, large quantities of secondary waste are generated resulting from the addition of chemical agents for precipitation and reduction in the processes. The biosynthetic method employing plant extracts have drawn attention as a simple and viable alternative to chemical and physical methods. the green seaweed *Calotropis procera* has been used as zinc oxide nanoparticles [4]. This biological approach appears to be a cost effective alternative to Conventional physical and chemical methods of synthesis.

Hence the present study was extract of green seaweed *Caulerpa peltata*, red *Hypnea Valencia* and brown *Sargassum myriocystum* three seaweeds were used for ecofriendly synthesis of zinc oxide nanoparticles. However, till date, there are no reports on the synthesis of zinc oxide nanoparticles with these seaweeds *Caulerpa peltata*, *Hypnea Valencia* and *Sargassum myriocystum* and very limited studies have been reported on the other seaweeds for biosynthesis of metal nanoparticles. The preliminary screening of three seaweeds by physico-chemical parameter such as concentration of metals, concentration of seaweed extract, temperature, pH and reaction time. This parameters were used to assess which seaweed can be rapid extracellular synthesis and suitable condition for synthesis of zinc oxide nanoparticles. The obtained zinc oxide nanoparticles were characterized by UV–vis spectroscopic analysis followed by, Dynamic light Scattering (DLS), Atomic Force Microscopy (AFM) measurements, Scanning Electron microscopy (SEM) and Energy dispersive X-ray analysis (EDAX), Transmission electron microscopy (TEM) and Fourier Transform Infrared Spectroscopy (FTIR). Based on the FTIR results, fucoidan water soluble pigments present in *S. myriocystum* leaf extract is responsible for reduction and stabilization of zinc oxide nanoparticles. This *S. myriocystum* is available in all season in the gulf of mannar, plenty and it is easy to make large scale production of ZnO nanoparticles. zinc oxide nanoparticles were obtained by using seaweed as both the reducing and stabilizing agent. Antibacterial activity was also demonstrated using the prepared nanoparticles on Gram-positive (*Staphylococcus aureus Streptococcus mutans)* and Gram-negative bacteria (*Vibrio cholerae, Neisseria gonorrohea,* and *Klebsiella pneumonia*). The antifungal activity against *Aspergillus niger* and *Candia sp.,* was also determined to find out the potential of the generated nanoparticles to provide effective natural nanomedicine active and against microbial infection.

## Result and discussion

### Screening and optimization of physico-chemical parameters of zinc nanoparticles

**Figure 1:**
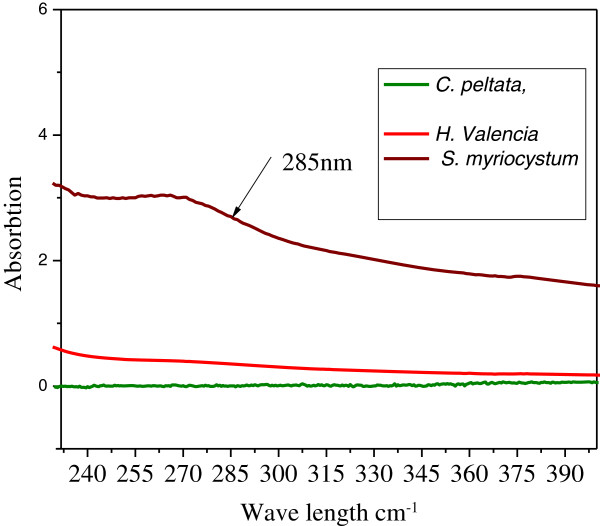
Effect of production of zinc nanoparticles by seaweeds.

Zinc oxide was intensive absorption in the ultraviolet band of about 300–500 nm. In this research a biosynthesis method was employed for preliminary evaluation of the reducing potential of three Seaweeds extracts by controlling different physico-chemical parameters. Since biological synthesis of metal and metal oxide NPs are focusing on controlled monodispersed, a number of seaweeds were screened for the study. Brown alga *P. tetrastromatica,* was the exciting candidate for the synthesis of silver NPs with stability [5]. Here too, brown alga *S. myriocystum* was found to be a good candidate for the synthesis of zinc oxide nanoparticles. Among the three seaweeds, UV study revealed the fact that the leaf extract of *S. myriocystum* exhibited rapid and stable synthesis of zinc oxide nanoparticles Figure 1.

### Effect of concentration

**Figure 2:**
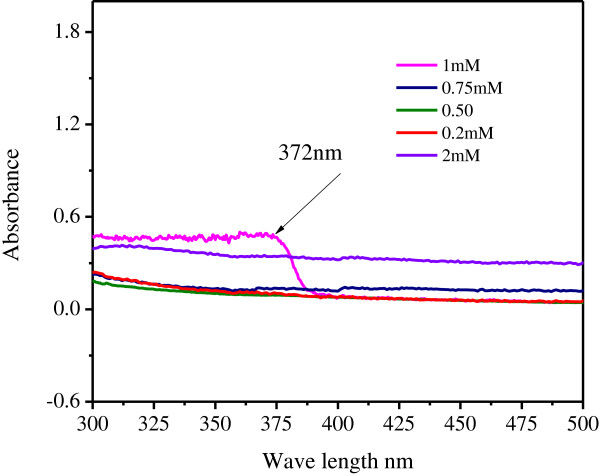
Effect of concentration on production of zinc nanoparticles.

By increasing the concentration of ZnNO_3_ from 0.25 to 2 mM and absorption peaks were studied. Figure 2 revealed that no absorption peaks were observed from 0.25- 0.75. Further it showed that 1 mM changes in UV–vis absorption indicated that the dispersion of Zinc nanoparticles was affected by the concentration, which operates as a controller of nucleation. The characteristic peak 372 nm were recorded for 1 mM zinc nitrate_._

### Effect of seaweed filtrate concentration

The effect of different leaves extract concentration of seaweed (5, 10, 15, 20 and 25 ml) was studied.

The use of low concentration of seaweed leaves extract are reacting with zinc led to the formation of increased absorbance at 380 nm. As soon as the extract concentration was increased beyond 25 ml, the solution started forming aggregates were formed. From the result, it was concluded that the optimum absorbance occurred at 5 ml of seaweed filtrate.

### Effect of temperature

**Figure 3:**
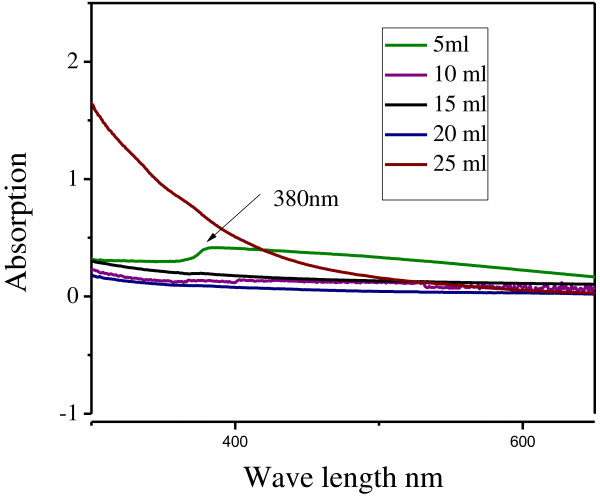
**Effect of *S. myriocystum *** **concentration production of zinc nanoparticles.**

To study the effect of temperature, 5 ml concentration of *S. myriocystum* leaves extract and concentration of 1 mM ZnNO_3_ samples were prepared. By increasing the temperature from 50 and100°C, results indicated that no absorption peak was observed in 50,60,70 and 90 and 100°C (Figure 3). It is seen that, at temperature 80°C the absorption peaks observed at 376 nm. This 80°C temperature the bulk zinc nitrate was converted to zinc nanoparticles.

### Effect of pH

**Figure 4:**
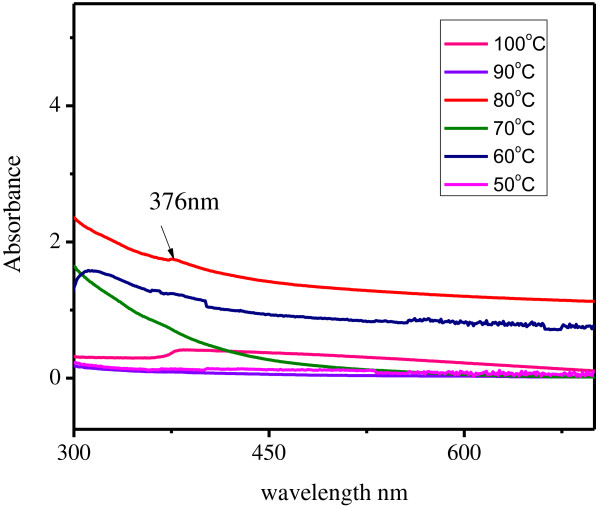
Effect of Temperature on production of zinc nanoparticles.

The lower pH 5–7 no absorption peak was observed in this region Figure 4. At low pH, the aggregation of zinc nanoparticles to form larger nanoparticles was believed to be favored over the nucleation. But in case of higher pH 9 and 10 no absorption peaks were observed. Likewise, absorption peak at pH 8 indicated the total reduction of zinc nitrate to zinc nanoparticles.

**Figure 5:**
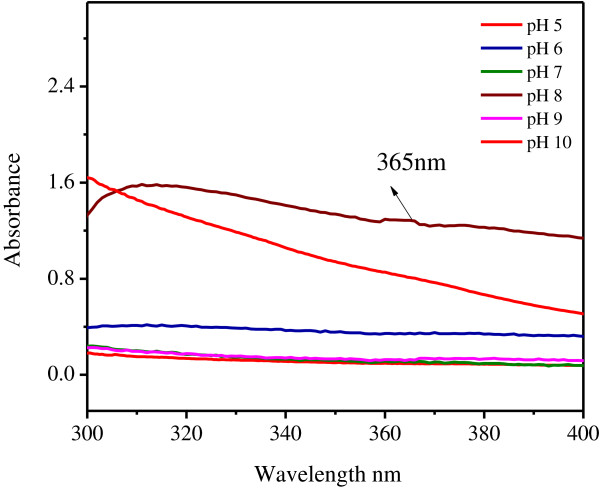
Effect of pH on production of zinc nanoparticles.

The absorption wavelength at about 372 nm of ZnO suggested the excitation blue shift character at room temperature. Blue shift is relative to presence of bulk ZnO nanoparticles [5]. Large plate structures could not be detected and most particles were spherical at leaf extract at 5 ml concentrations at 380 nm (Figure 5). The particle size was observed to increase with an decrease in the leaf broth concentration. The use of a high concentration of the *S. myriocystum* extract reacting with zinc nitrate led to the formation of hexagonal, triangular, rod and radial nanoparticles, while the shape of the nanoparticles changed to spherical on decreasing the concentration of the *S. myriocystum* leaves extract. Similar changes in the shapes and size were observed on assisted zinc nanoparticle *Calotropis procera* biosynthesis [4].

*S. myriocystum* extract concentration in the 5.0 ml 1 mM bulk zinc nitrate and reaction duration from 10 to 30 min at pH 7. The other parameter used for control synthesis of metal nanoparticle was temperature. The characteristic absorption peak 376 nm was observed at 80°C (Figure 3). The zinc nanoparticle formation depends on nucleation and growth mechanism. At low temperature rate of growth controls the size of the particles, while at higher temperature number of nuclei formed will increase and hence small particle size is observed [6].

At low pH, the aggregation of zinc nanoparticles to form larger nanoparticles was believed to be favored over the nucleation. From Figure 1 at pH 8, absorption peak was observed at 365 nm. It clearly indicate higher pH has favored higher reducing power [7]. Reported reduction process took place in two stages with an optimum pH range of 4–9 with a maximum uptake obtained at pH 7. Biosynthesis controlled by the pH of the solution used during the gold bioreduction process.

**Figure 6:**
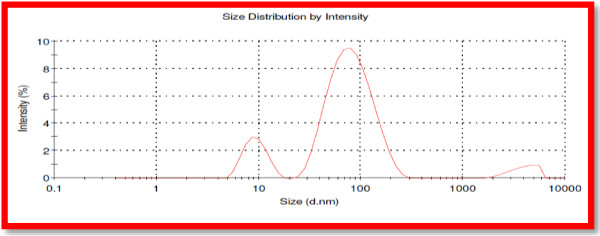
Particle size distribution of Zn nanoparticles by DLS.

The results of the particle size distribution (PSD) dynamic light scattering method conforming the presence of zinc oxide nanoparticles, particle Z-Average size as 46.61 nm and Poly dispersity index as 0.552 (Figure 6).

From the Figure 6 it was clear that the solution contained zinc nanoparticles having average particle size of 46.61 nm with poly dispersity index of 0.552. It clearly indicates that the obtained zinc oxide nanoparticles are monodispersed in nature. Samples with very broad size distribution have polydispersity index values > 0.7. These findings also ascertained the monodispersed zinc oxide nanoparticles as suggested by Anilreddy [8]. The result was in accordance with SEM data.

### Atomic force microscope analysis

**Figure 7:**
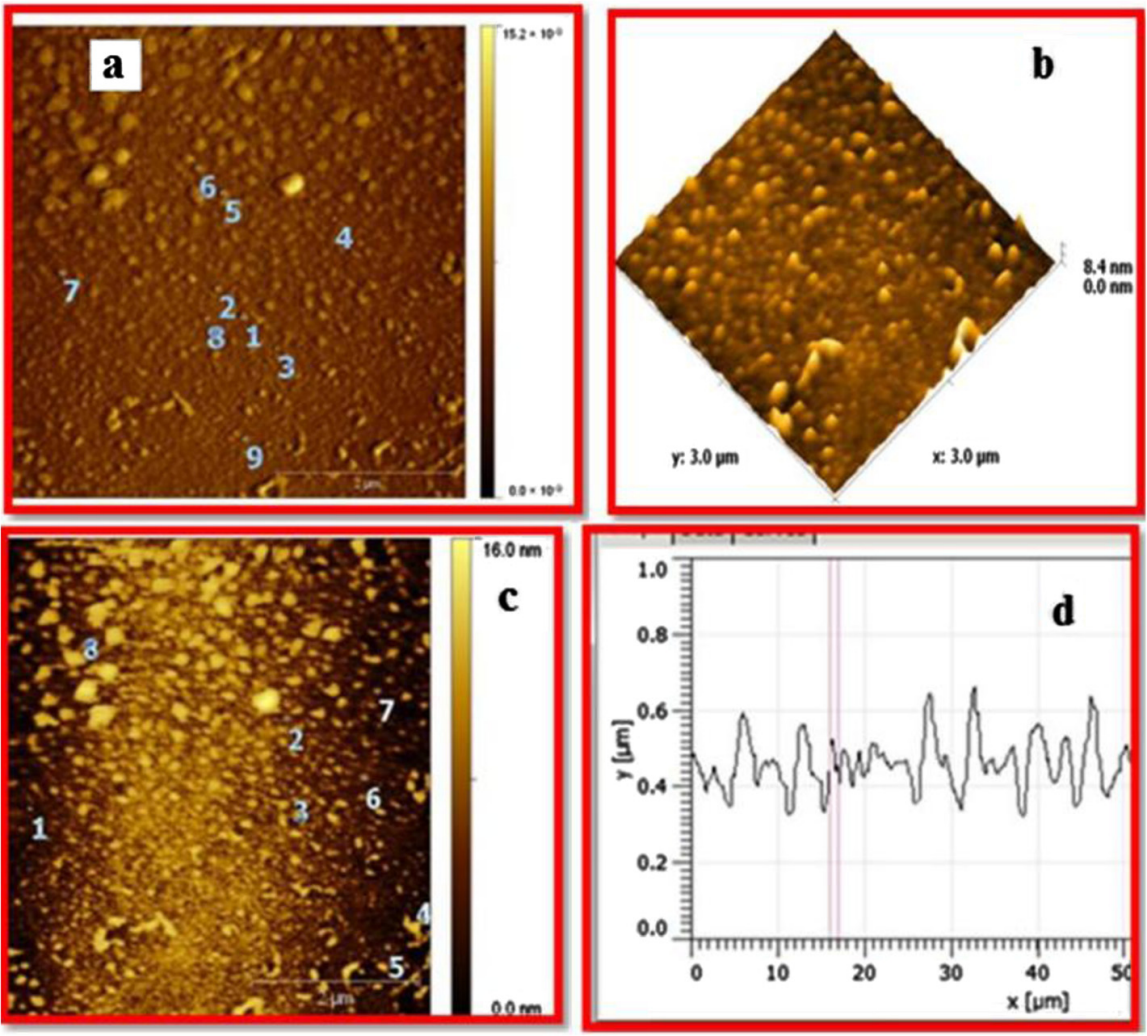
**AFM results of zinc oxide nanoparticles 2D and 3D images. (a)** Unfiltered AFM image showing topographical 2D image of zinc nanoparticles **(b)** 3D image of synthesized **(c)** Filtered 2D AFM image of zinc nanoparticles **(d)** particle size distribution of zinc nanoparticles.

The size of the nanoparticles is obtained directly from tip-corrected AFM measurements, and the shape of the nanoparticle is estimated on the basis of AFM images and line scans. The tip-corrected measured nanoparticles 20–36 nm (Figure 7). The results show two and three dimensional view of sample surface over a 2 × 2 μm scan and uniform height distribution around 8.4 nm (Figure 7). Similarly, result show filtered 2D AFM image of zinc oxide nanoparticles using line profile (Figure 7). The result of Line profile images of single zinc oxide nanoparticle was 36 nm (Figure 7).

AFM analysis of zinc oxide nanoparticles indicated that the change of parameter temperature greatly affected the morphology and size of the particles. TEM result of zinc oxide nanoparticles showed 76–186 nm. In fact, the increase in reaction temperature to 90°C, resulted in a significant decrease in particle size of zinc oxide nanoparticles from 76-186 nm to 20 to 36 nm (Figure 7). The result of size distribution was also confirmed through XRD analysis. The mono dispersed single zinc oxide nanoparticles size measured by AFM line profile was found to be 36 nm.

### SEM and EDX analysis

SEM has been used to examine the surface morphology and to estimate the obtained structural rectangle, triangle, radial hexagonal, rod and spherical shapes.

**Figure 8:**
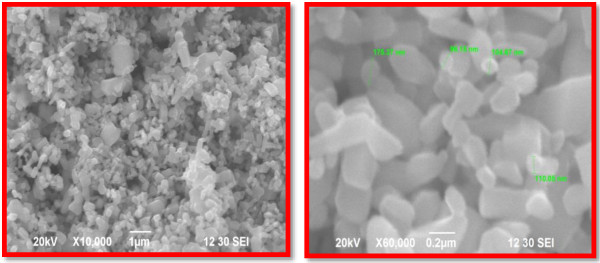
**SEM image of the synthesized zinc nanoparticles.** The SEM image of Zn nanoparticles, reveals its size as 96-110 nm (Figure 8). Figure 9 shows the EDS analysis of ZnO nanoparticles 52% of zinc and 48% of Oxides which confirms the elemental composition of ZnO nanoparticles.

**Figure 9:**
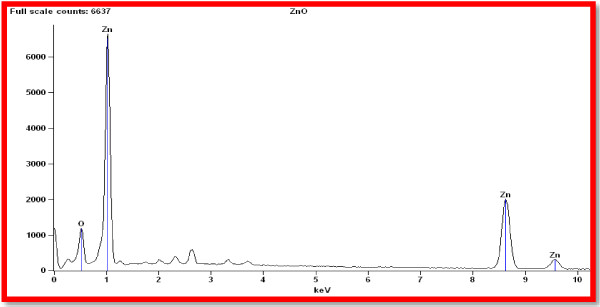
EDX Spectrum of ZnO nanoparticles.

The SEM image of Zn nanoparticles, reveals its size as 96-110 nm (Figure 8). Figure 9 shows the EDS analysis of ZnO nanoparticles 52% of zinc and 48% of Oxides which confirms the elemental composition of ZnO nanoparticles.

Typical SEM micrographs image of the ZnO nanoparticles size 96-110 nm obtained by the biosynthesis method. The shapes of Zinc oxide nanoparticles were rectangle, spherical, triangle, radial and spheres. The resulting zinc oxide nanoparticle morphology was quite similar to the shape obtained through TEM analysis. EDX spectrum shows four peaks which were identified as zinc (52%) and oxygen (48%) (Table 1). The EDS analysis display the optical absorption peaks of ZnO nanoparticles and these absorption peaks were due to the surface plasmon resonance of Zinc oxide nanoparticles. The origin of these elements lies in the biological components, mostly algin along with Zn O nanoparticles [9].

**Table 1:** Elemental composition of ZnO nanoparticles

**Element**	**Weight %**	**Atom %**
Zn	20.15	51.61
O	79.85	48.39
Total	100	100

### Transmission electron microscope analysis

In order to determine the structure of the nanoparticles, transmission electron microscopy (TEM) was used.

**Figure 10:**
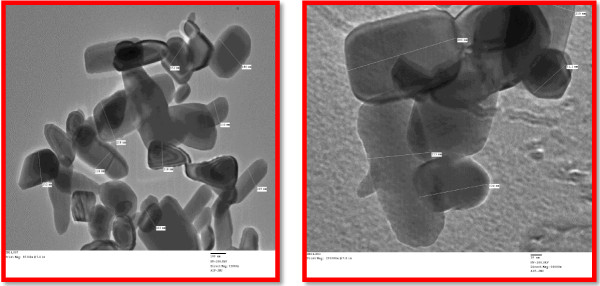
TEM results of biosynthesized zinc oxide nanoparticles.

Most of the zinc oxide nanoparticles falls within the range of different sizes are triangle, radial, hexagonal, rod, and rectangle sizes 76- 186 nm Figure 10. A clear TEM image of ZnO NPs was generated at pH 7. Most of the zinc oxide nanoparticles shapes were spherical, triangle, radial, hexagonal, rod and rectangle with size 76 - 186 nm. TEM images of representative nanoparticles show that the nanoparticles, appear higher than the estimated results from Scherrer analysis (lesser size 36 nm). pH 8 was a good pH to investigate size-dependent behavior due to the fact that there is minimal interference. Once again in the biosynthesis of ZnO nanoparticles using *S. myriocystum* the pH was increased from 7 to 8. But the concentration of *S.myriocystum* was kept constant [10] also reported that zinc oxide dissolution is well-known and occurs over a wide range of pHs. The present study agrees with [11] concluding that the citric acid enhanced the extent of ZnO dissolution for all sizes, and the greatest enhancement was observed in zinc oxide particles.

### X-ray diffraction analysis

**Figure 11:**
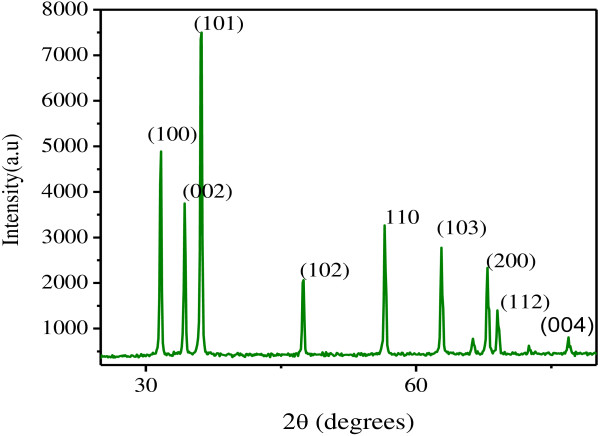
**X-ray diffraction pattern at 2θ: 30**^**о**^**– 90**^**о **^**for Zn O nanoparticles.**

The phase purity and composition of the products obtained by the biosynthesis using extract of *S. myriocystum* examined by XRD*.* Figure 11 shows a typical XRD pattern of ZnO nanoparticles in the range of 20 – 80° at a scanning step of 0.01.

A number of Bragg reflections with 2θ values of 31.6°, 34.35°, 36.2°, 47.5°, 56.4°, 62.8°, 67.8°, 68.9° and 76.9° are observed corresponding to (100), (002), (101), (102),(110), (103), (200), (112) and (004) planes, shows a typical XRD pattern of ZnO nanoparticles in the range of 30 – 80° at a scanning step of 0.01 (JCPDS card No 89–1397). Average size of the zinc oxide nanoparticles was determined as 36 nm from the width of the dominant peak (101) reflection according to the Debye–Scherrer equation. The XRD pattern thus clearly illustrates that the ZnO nanoparticles nanoparticle synthesized by the green method. All diffraction peaks are indexed according to the hexagonal phase of ZnO. No characteristic peaks of impurity phases of nitrate and other impurity except ZnO are found which revealed that good crystalline in nature of the samples.

The XRD pattern thus clearly illustrated that the ZnO nanoparticles synthesized by the green method were spherical and crystalline in nature. It was quite common, the broadening of the peaks in the XRD patterns of solids is attributed to particle size effects [12]. Average size of the zinc oxide nanoparticles was determined from the width of the reflection according to the Debye–Scherrer equation: D = (0.9 l/λ)/(β cos *Ө*), where β is the full width at half maximum (FWHM) of the peak in radians, λ is the angle of diffraction and λ is the wavelength of the X-ray. By considering the FWHM of the dominant diffraction peak (101) of zinc oxide nanoparticles, the crystalline size of the ZnO was calculated as 36 nm at pH 8.

### Analysis of chemical nature before and after synthesis

#### FTIR analysis

The bands of biosynthesized zinc nanoparticles from *S. myriocystum* were noticed at 3409, 2366, 1736 1073 and 442 cm^-1^ in the FTIR spectrum, Whereas, bands of *S.myriocystum* extract were noticed at 3465, 2388,1652 and 1070 cm^-1^.

The intense broad band at 3409, 2366 cm^-1^ can be assigned to O-H and C = O stretching band. The band at 3465, 2388, 1652 and 1070 cm^-1^ of *S. myriocystum* extract shifted to 3409, 2366, 1736,1023 and 442 cm^-1^ The absorption band corresponding to 3409 cm^-1^ was due to C-H, stretching vibrations of carboxylic acid and hydroxyl stretch vibrations. Further, 1736 cm^-1^ representing to C = O carboxylic acid and the strong C-H group bonds at 1023 cm^-1^, which are sharper and broader for zinc oxide nanoparticles participates in the reaction.

**Figure 12:**
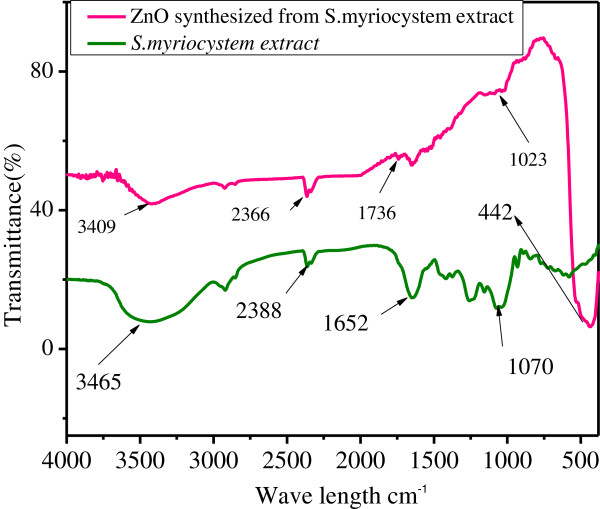
**FTIR-Transmittance spectra of *S. myriocystum extract *** **and biosynthesized of Zinc oxide nanoparticles.**

In Figure 12, IR spectra peak shows the bulk ZnO showing a high intensity broad band around 442 cm^-1^ due to the stretching mode of the zinc and oxygen bond [13-15]. The significant changes might be due to complexes of zinc with the sites. The remaining peaks 3465, 2388, 1652 and 1070 cm^-1^ were correspond to *S. myriocystum* extracts*.* The leaves extract of *S. myriocystum* showed peaks at 3465, 2388, 1652 and 1070 cm^-1^ along with other weak bands. The sharp pointed peak observed, 1070 cm^-1^, corresponds to hydroxyl group.

These peaks occurred due to *S. myriocystum* which is enriched with photochemical such as alginic acid, ascorbic acid, protein, carbohydrates, flavanoids, tannins, mannitol and lipids [16-18]. These soluble elements could have acted as both reduction and stabilizing agents preventing the aggregation of nanoparticles in solution, extracellular biological synthesis of zinc oxide nanoparticles size 36 nm.

### Antimicrobial activity

The metal nanoparticles of zinc oxide exhibited moderate activity against *S. mutans* gram positive bacteria. The nanoparticles of exhibited weak activity against *M. luteus.* The metal nanoparticle of zinc exhibited moderate activity against *V. cholerae* the negative bacteria, but *K. pneumoniae* had weak activity, and *N. gonorrohea* showed strong activity. The significant antimicrobial activity of the metal nanoparticles was comparable to the standard antibiotics Penicillin (10 μg/ disk) and Amphotericin (10 μg/ disk) (Tables 2 and 3).

**Table 2:** Antimicrobial activity of metal nanoparticles against Gram positive bacteria

**Metal nanoparticles**	***S. mutans***	***M. luteus***
Zinc	++	+
Penicillin (Antibiotics)	–	–
Amphotericin (Antibiotics)	+	+

–, No activity; +, weak activity (<5 mm zone of inhibition); ++, moderate activity (>5 mm zone); +++, strong activity (>10 mm zone).

**Table 3:** Antimicrobial activity of metal nanoparticles against Gram negative bacteria

**Metal nanoparticles**	***V.cholerae***	***K. pneumoniae***	***N. gonorrohea***
Zinc	++	+	+++
Penicillin (Antibiotics)	–	–	_
Amphotericin (Antibiotics)	+	+	+

The presence of reactive oxygen species (ROS) generated by ZnO nanoparticles is responsible for their bactericidal activity. Further, it showed that the antibacterial behavior of ZnO nanoparticles could be due to chemical interactions between hydrogen peroxide and membrane proteins, or between other chemical species produced in the presence of ZnO nanoparticles and the outer lipid bilayer of bacteria. The hydrogen peroxide produced enters the cell membrane of bacteria and kills them. It was shown in the study that nano - sized ZnO particles are responsible for inhibiting bacterial growth [19]. Zinc oxide exhibited strong antimicrobial activity against *C. albicans*, whereas it exhibited moderate activity *A. niger* against (Table 4)*.* Reports of Lipovsky [20] supported the finding that zinc oxide nanoparticles provide a novel family of fungicidal compounds and histidine suggest the involvement of reactive oxygen species, including hydroxyl radicals and singlet oxygen, in cell death.

**Table 4:** Antimicrobial activity of metal nanoparticls against fungi

**Metal nanoparticles**	***C. albicans***	***A. niger***
Zinc	+++	++
Penicillin (Antibiotics)	–	–
Amphotericin (Antibiotics)	+	+

## Conclusion

In the present study by using seaweed *S. myriocystumz* ZnO was rapidly biosynthesized at pH 8 and the size of nanoparticles was 36*.* TEM analysis show zinc oxide nanoparticles of different shapes viz., spherical, triangle, radial, hexagonal, rod, and rectangle size 76-186 nm. After changing temperature mono dispersed single zinc oxide nanoparticles size 36 nm was measured by AFM line profile and FWHM of the dominant (113) diffraction peak of Iron oxide nanoparticles, the crystalline size of the ZnO was calculated as 36 nm. IR spectra peak 442 cm^-1^ indicated characteristic absorption bands of ZnO nanoparticles. Silva, 2002 [14] based on the FTIR results, fucoidan water soluble pigments present in *S. myriocystum* leaf extract is responsible for reduction and stabilization of metal and metal oxide nanoparticles. Thus this method appears to be a potentially exciting tool for large-scale synthesis of nanoparticles. The findings also revealed that brown seaweeds have the natural potential for the synthesis of nanoparticles and are regarded as potential biofactories for nanoparticles synthesis. zinc oxide nanoparticles can be used in effluent treatment process for reducing microbial load.

## Materials and methods

### Preparation of the seaweed extraction

**Figure 13:**
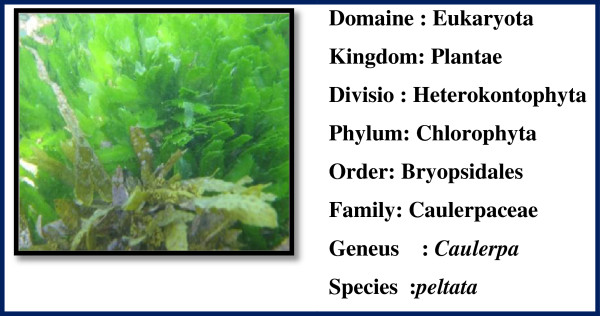
**Morphology of green seaweed *caulerpa peltata.***

**Figure 14:**
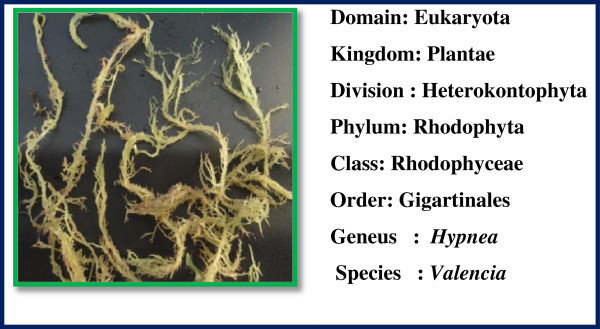
**Morphology of red seaweed *hypnea valencia.***

**Figure 15:**
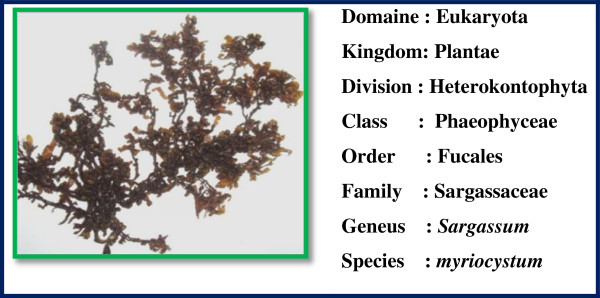
**Morphology of brown seaweed *sargassum myriocystum.***

Fresh and healthy *Caulerpa peltata*, *Hypnea Valencia* and *S. myriocystum* (Figures 13, 14 and 15) Seaweeds were collected from intertidal rocky shore regions in Mandapam, Pudhumadam and Kilakarai coast of the Gulf of Mannar region, India keys available [21]. Collected seaweeds were washed thoroughly with distilled water, incised into small pieces and air-dried. About 10 g of finely cut into small pieces were weighed and transferred into 500-ml beaker containing 100 ml distilled water, mixed well and boiled for 25 min. The extract obtained was filtered through Whatman No.1 filter paper and the filtrate was collected in a 250-ml Erlenmeyer flask and stored in refrigerator at 4°C for further use. This process was repeated for each seaweed sample separately to collect the filtrate.

### Biosynthesis of zinc nanoparticles

For the preparation of extracellular synthesis, 5 ml of seaweed leaf extract was added 95 ml of aqueous solution of 1 mM zinc nitrate. Then the solution was kept under constant stirring using a magnetic stirrer to completely dissolve the zinc nitrate. After complete dissolution of zinc nitrate, the flask containing the solution was heated on a water bath at 80°C for 5–10 min. The pH was adjusted between 5 and 10 using 0.1 M HCl or 0.1 M NaOH aqueous solution to the above aqueous solution and placed on magnetic stirrer for 1 h. This procedure was applied to all three different seaweed filtrates. The spectra exhibit an absorption band with a resolution of 1 nm between at 300 nm and 600 nm.

### Optimization of physico-chemical parameters for metal nanoparticle biosynthesis

Different parameters were optimized including concentration of metal, concentration of seaweed filtrate, reaction temperature, pH and time which had been identified as factors affecting the metal nanoparticles formation. These optimization parameters were maintained as controller of biological synthesis of metal nanoparticles.

### Effect of metal ion concentration

The concentrations of metals were maintained at 0.25, 0.5, 1.0 and 2.0 mM. The concentration of seaweed and pH 8 of the resulting solution were kept constant. The absorbance values of the resulting solutions were measured spectrophotometrically.

### Effect of seaweed leaf filtrate concentration

The effect of change in seaweed leaf filtrate solution of the reaction mixture was analysed for determining the rapid synthesis of metal nanoparticles. The different concentrations of leaf filtrate were maintained as *viz*., 5, 10, 15, 20 and 25 ml. The metal ion concentration (1 mM) of metal, temperature (80°C) and pH (8) of the reaction mixture were kept constant. The absorbance of the solution was measured spectrophotometrically.

### Effect of reaction temperature

The effect of temperature on the reaction medium was investigated for the optimum synthesis of three metal nanoparticles. After Extracellular biosynthesis, the important physico-chemical parameter temperature was maintained at 50, 60, 70, 80, 90 and 100°C using a water bath. The metal solution concentrations and aqueous extract from 5 g of seaweeds were kept constant. The absorbance of the resulting solutions was measured spectrophotometrically.

### Effect of pH

pH was maintained at 5, 6, 7, 8, 9 and 10 which was adjusted using 0.1 M HCl or 0.1 M NaOH; duration of reaction time was maintained from 1 h to 72 h. The above mentioned procedure was repeated to optimize the time required for the completion of reaction, where the reaction was monitored from 0 to 60 min. The concentration of seaweed and temperature 80°C of the resulting solution were kept constant. The absorbance of the resulting solutions was measured spectrophotometrically.

### Effect of reaction time

The stability of the resultant solution was determined at room temperature, at intervals of 1 h, 24 h, 72 h and 1, 2 and 6 months.

### Purification of nanoparticles

#### *Centrifugation and lyophilization of metal nanoparticles*

To remove the non-metal components along with a maximal recovery of metal nanoparticles from the synthesized solution, an optimal centrifugation process was obtained based on tests of two centrifugation forces 12,000 and 15000 rpm for 30 min. Biosynthesized metal particles in aqueous upper layer were collected. Collected layer of solution was redispersed in sterile deionized water to get rid of any biological molecules. The process of centrifugation and redispersion in sterile deionized water was repeated thrice to obtain better separation of entities from the metal nanoparticles. The centrifugation process was done using refrigerated centrifuge (REMI, India). The purified solution of metal nanoparticles were then freeze dried using a lyophilizer (Micro Modulyo 230 freeze dryer, Thermo Electron Corporation, India). Then dry powder was mixed with 10 ml deionised water and kept on a sonicator to prevent aggregation of ions.

### Characterization of metal nanoparticles

The synthesized nanoparticles were characterized by various techniques viz., UV–vis Spectroscopy analysis, Dynamic light Scattering(DLS), Atomic Force Microscopy (AFM) measurements, Scanning Electron microscopy (SEM) and Energy dispersive X-ray analysis (EDAX), Transmission electron microscopy (TEM) and Fourier Transform Infrared Spectroscopy (FTIR) which provide important information for the understanding of different physicochemical features.

Then pellet was mixed with 10 ml deionised water and kept on sonicator for prevent aggregation of zinc ions. The dried powder was analyzed using FTIR (model no 8400 s SHIZAMAZU). A disk of 1:3 ratio of KBr was prepared with mixture of dried samples and then examined under IR-Spectrometer. Infrared spectra were recorded in the region of 500 to 4000 cm^-1^. The bioreduced zinc oxide Nanoparticle solution was drop-coated onto glass substrate and powder X-ray diffraction measurements were carried out in an (Shimadzu- model XRD 6000) X-ray diffractometer. The pattern was recorded by Cu K α radiation with λ of 1.54 Å. The lyophilized zinc oxide Nanoparticles were mounted on carbon stubs and the images were studied using scanning electron microscope (SEM-EDX JEOL Model-L6390). The size and elemental status of zinc oxide NPs were confirmed by SEM EDX. AFM images were collected using a Digital Instruments Nanoscope microscope at 27°C. Etched Si nitrate nanoprobe tips (APE Research- model no: A100SGS) were used. These tips have spring constants of approximately 0.15 nm^-1^ and are conical in shape with a cone angle of 20° and an effective radius of curvature of 10 nm at the tip. The size and 3D structure nanoparticles were confirmed by AFM.

### Antimicrobial activity

The synthesized nanoparticles were tested for antibacterial activity by agar disk-diffusion method against human pathogenic bacteria *viz*., the gram positive bacteria such as *Streptococcus mutans* and *Staphylococcus aureus* and the gram negative bacteria *Vibrio cholerae, Neisseria gonorrohea,* and *Klebsiella pneumonia and* fungal cultures *Aspergillus niger* and *Candida albicans.* Each strain was individually swabbed uniformly onto the surface of Mueller Hinton agar plate using sterile cotton swab (Himedia Labs, Mumbai, India). 1 mg of synthesized nanoparticles was dissolved in 1 ml of de-ionized water. Different concentrations of aqueous solution of nanoparticles in solution (10 μl, 20 μl, 40 μl) were poured onto each disk and placed on Mueller Hinton agar plates. The two different drugs used in this study were Penicillin and Amphotericin.

## Abbreviations

2D: Two dimension; 3D: Three dimension; AFM: Atomic force microscope; cm: Centimeter; DLS: Dynamic light scattering; EDX: Energy dispersive x-ray analysis; eV: Electron volt; Fig.: Figure; FTIR: Fourier transform infrared spectroscopy; FWHM: Full width of half maximum; g: Gram; HCL: Hydrochloric acid; IR: Infrared; M: Molar; min: Minute; ml: Milliliter; mM: Milli molar; NaOH: Sodium hydroxide; nm: Nanometer; NPs: Nanoparticles; PDI: Polydispersity index; Rpm: Revolutions per minute; SEM: Scanning electron microscope; SPR: Surface plasmon resonance; TEM: Transmission electron microscopy; Vis: Visble; XRD: X-ray diffraction; ZnO: Zinc oxide.

## Competing interests

The authors confirm that this article content has no competing interests.

## Authors’ contributions

NS carried out biosynthesis and all characterization. AKK sir conceived of the study, and participated in its design and coordination and helped to draft the manuscript. Both authors read and approved the final manuscript.
